# Investigation of PAS and CNBH domain interactions in hERG channels and effects of long-QT syndrome-causing mutations with surface plasmon resonance

**DOI:** 10.1016/j.jbc.2021.101433

**Published:** 2021-11-19

**Authors:** Stephanie M. Soohoo, Purushottam B. Tiwari, Yuichiro J. Suzuki, Tinatin I. Brelidze

**Affiliations:** 1 Department of Pharmacology and Physiology, Georgetown University Medical Center, Washington, District of Columbia, USA; 2 Department of Oncology, Georgetown University Medical Center, Washington, District of Columbia, USA

**Keywords:** KCNH, EAG, ELK, Kv11.1 channels, KCNH1, KCNH2, KCNH4, potassium channel, LQTS, CNBH, C-terminal cyclic nucleotide-binding homology, HCN, hyperpolarization-activated cyclic nucleotide-gated, hERG, human *ether-á-go-go-related* gene, LQTS, long-QT syndrome, PAS, Per-Arnt-Sim, SPR, surface plasmon resonance, TEV, tobacco etch virus

## Abstract

Human *ether-á-go-go-related* gene (hERG) channels are key regulators of cardiac repolarization, neuronal excitability, and tumorigenesis. hERG channels contain N-terminal Per-Arnt-Sim (PAS) and C-terminal cyclic nucleotide-binding homology (CNBH) domains with many long-QT syndrome (LQTS)-causing mutations located at the interface between these domains. Despite the importance of PAS/CNBH domain interactions, little is known about their affinity. Here, we used the surface plasmon resonance (SPR) technique to investigate interactions between isolated PAS and CNBH domains and the effects of LQTS-causing mutations R20G, N33T, and E58D, located at the PAS/CNBH domain interface, on these interactions. We determined that the affinity of the PAS/CNBH domain interactions was ∼1.4 μM. R20G and E58D mutations had little effect on the domain interaction affinity, while N33T abolished the domain interactions. Interestingly, mutations in the intrinsic ligand, a conserved stretch of amino acids occupying the beta-roll cavity in the CNBH domain, had little effect on the affinity of PAS/CNBH domain interactions. Additionally, we determined that the isolated PAS domains formed oligomers with an interaction affinity of ∼1.6 μM. Coexpression of the isolated PAS domains with the full-length hERG channels or addition of the purified PAS protein inhibited hERG currents. These PAS/PAS interactions can have important implications for hERG function in normal and pathological conditions associated with increased surface density of channels or interaction with other PAS-domain-containing proteins. Taken together, our study provides the first account of the binding affinities for wild-type and mutant hERG PAS and CNBH domains and highlights the potential functional significance of PAS/PAS domain interactions.

The human *ether-á-go-go-related gene* (hERG) channels, also known as Kv11.1 and KCNH2, are voltage-gated potassium channels that generate rapidly activating, delayed rectifier K^+^ currents (I_Kr_) in the heart (1, 2). I_Kr_ currents are key contributors of the ventricular action potential repolarization due to the large hyperpolarizing currents generated during the slow deactivation (closing) of hERG channels (3, 4). Over 100 mutations in hERG channels have been linked to inherited cardiac arrhythmias, such as long QT syndrome (LQTS) (5). In addition to the heart, hERG channels are expressed in the brain where they regulate neuronal excitability, and changes in hERG channel currents contribute to increased risk of schizophrenia (6, 7, 8, 9). hERG channels are also frequently overexpressed in cancer, and inhibition of hERG currents has been shown to decrease cancer progression (10, 11, 12).

hERG channels belong to the KCNH family of potassium channels, which also includes *ether-á-go-go* (EAG) and EAG-like (ELK) channel subfamilies (13). KCNH channels are assembled by four subunits, each composed of six transmembrane segments (S1–S6) and intracellular N- and C-termini (Fig. 1*A*) (13, 14, 15). Transmembrane segments S1 to S4 form the voltage-sensor domain and S5 to S6 segments form the centrally located pore domain. The N-terminus contains a Per-Arnt-Sim (PAS) domain, with the first 25 amino acids of the domain forming a PAS-cap region. The C-terminus contains a cyclic nucleotide-binding homology (CNBH) domain linked to the S6 transmembrane segment *via* the C-linker. The C-linker/CNBH domains ring the intracellular pore entrance with the PAS domains located at the periphery of the tetrameric ring assembly (Fig. 1*B*). Despite the structural homology to the cyclic nucleotide-binding domains in hyperpolarization-activated cyclic nucleotide-gated (HCN) channels, CNBH domains in KCNH channels do not function as cyclic nucleotide-binding domains (16, 17). Instead of cyclic nucleotides, the putative cyclic nucleotide-binding site in the CNBH domain of KCNH channels is occupied by a short stretch of amino acids, called the intrinsic ligand (Fig. 1*B*) (18, 19, 20).

**Figure 1:**
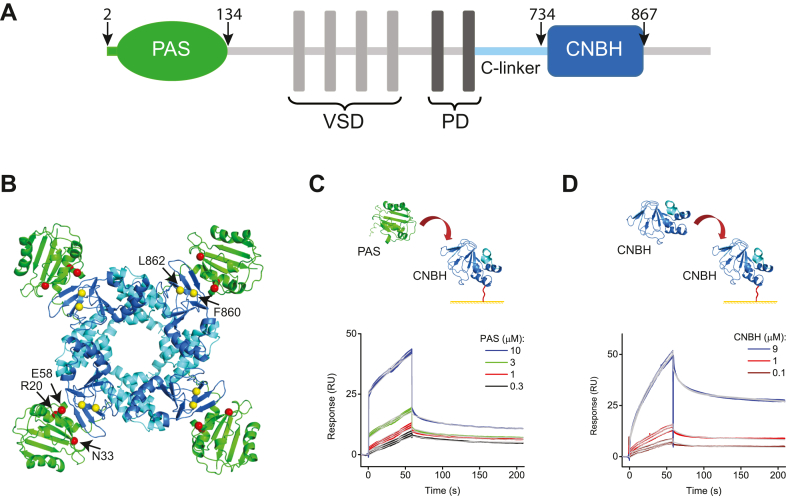
**hERG PAS and CNBH domain interactions detected with SPR.***A*, a linear representation of hERG channel topology. The *green line* and *green oval* represent the N-terminal PAS-cap and PAS domain, respectively. *Light gray vertical rectangles* represent the voltage sensor domain (VSD) and *dark gray rectangles* represent the pore domain (PD). *Cyan line* and *blue rectangle* represent the C-linker and CNBH domain, respectively. The *arrows* and *numbers* indicate the first and last residues of the PAS and CNBH domain constructs used in the study, respectively. *B*, a ribbon representation of the tetrameric assembly of the intracellular PAS and C-linker/CNBH domains. PDB ID 5VA2. The same color coding as in (*A*). The intrinsic ligand residues F860 and L862 and three residues at the PAS/CNBH domain interface, R20, N33, and E58, mutations in which cause LQTS are shown in *yellow* and *red spheres*, respectively. *C*, schematic of the hERG PAS domain applied at the indicated concentrations to the hERG CNBH domain immobilized on the CM5 sensor chip and representative SPR sensorgrams. *D*, schematic of the hERG CNBH domain applied at the indicated concentrations to the hERG CNBH domain immobilized on the CM5 sensor chip and representative SPR sensorgrams. *Gray lines* in (*C* and *D*) represent fits of the data with the two-state reaction binding model using the Biaevaluation software. K_d_ values are 1.4 μM and 5 μM for sensorgrams in (*C* and *D*), respectively.

The intracellular domains of hERG channels exert functional effect *via* several important interactions. Firstly, functional and structural studies showed that PAS domains interact with C-linker/CNBH domains from adjacent subunits, and this interaction confers the hallmark slow deactivation of hERG channels that is essential for the repolarization of cardiac action potential (14, 15, 21, 22, 23, 24, 25, 26, 27, 28, 29). The PAS/C-linker-CNBH interdomain interaction interface is quite extensive and is formed by three networks of interactions: a network of interactions between the PAS-cap and C-linker, interactions between the intrinsic ligand and the PAS domain, and the more dispersed interaction network formed by the cores of the PAS and CNBH domains (14, 24, 30). Many of genetically occurring LQTS-causing mutations are located at the interface between the PAS and C-linker/CNBH domains (5, 14, 24, 31). Secondly, C-linker/CNBH domains from adjacent subunits interact, and this interaction leads to the formation of the intracellular tetrameric ring assembly (Fig. 1*B*). The interaction interface is largely formed by the C-linker resides, similar to the “elbow-on-shoulder” interactions initially observed in the cyclic nucleotide-binding domains of HCN channels (32, 33). Finally, there is evidence that the PAS domains of KCNH channels could form oligomers (34). While PAS domains on different subunits are too far apart to interact within a single hERG channel (Fig. 1*B*), potential interactions between the PAS domains of neighboring hERG channels or between hERG PAS and PAS-domain-containing non-KCNH proteins might be physiologically relevant.

Although the structural and functional studies highlight the importance of PAS and CNBH domains and interactions between them for hERG channel gating, the affinity of these interactions is not known. Here, we used surface plasmon resonance (SPR) technique to directly investigate interactions between the isolated PAS and CNBH domains of hERG channels. Using this approach, we determined the affinity of PAS/CNBH and CNBH/CNBH domain interactions. We found that the double mutation F860G/L862G in the intrinsic ligand and LQTS-causing mutations R20G and E58D located at the interdomain interface had no effect on the PAS/CNBH domain binding affinity, while N33T mutation, also located at the interdomain interface, and high ionic strength abolished PAS/CNBH domain interactions. We also determined the affinity of PAS/PAS domain interactions with SPR and using electrophysiology showed that coexpression of hERG channels with the isolated PAS domain in *Xenopus laevis* oocytes or injection of the purified PAS domain protein into hERG channel expressing oocytes resulted in a dramatic decrease in hERG currents, suggesting that the PAS/PAS domain interactions can affect hERG channel function. To the best of our knowledge, our findings provide first look at the affinity of the interdomain interactions in hERG channels, the effect of the mutations in the residues at the PAS/CNBH domain interface on the interdomain interactions and highlight the potential importance of PAS domain oligomerization for hERG-channel-dependent cellular signaling.

## Results

### Interactions between PAS and CNBH domains probed with SPR

To investigate PAS and CNBH interdomain interactions in hERG channels, we purified PAS and CNBH domains and used standard amine coupling chemistry to immobilize CNBH domains on a CM5 sensor chip (Fig. 1, *C* and *D*). Due to the limitations with purifying concentrated PAS and CNBH domains in volumes necessary for the SPR experiments, the maximal concentration for free domains used in our study was 10 μM. The CNBH domain used in our study contained the same stretch of amino acid as the protein used to determine the recently published high-resolution crystal structure of the isolated CNBH domain of hERG channels (35). In addition to the amino acids forming the CNBH domain, residues 748 to 867, the construct used in our study also contained the last 14 amino acids of the C-linker. Structures of the isolated PAS and CNBHD domains of hERG channels are very similar to the structures of the corresponding domains in the full-length hERG cryo-EM structure (Fig. S1) (14, 26, 35). Therefore, although the SPR experiments are carried out using the isolated domains, our findings should be relevant to the interdomain interactions in the context of the full-length hERG channels.

We applied free PAS domains over the range of concentrations to the immobilized CNBH domains (Fig. 1*C*). The SPR response increased with the increase in the PAS domain concentration. To determine the interaction affinity between the PAS and CNBH domains, the SPR response profiles were fitted with the two-state reaction model, using Biaevaluation software version 1.0, as predicted using procedures described in a previous publication (36) and described in the Experimental procedures section. The association and dissociation rate constants determined from the fits over the range of the tested PAS domain concentrations were used to calculate the affinity of the PAS and CNBH domain binding. The fitting of the SPR response profiles revealed the averaged binding affinity for PAS and CNBH domains of 1.4 ± 0.6 μM (Table 1).

**Table 1:** Summary of binding affinities (K_d_ in μM) for PAS and CNBH domain interactions

Injected domain (analyte)	Immobilized domain		
	CNBH	PAS	CNBH-FL/GG
CNBH	2.7 ± 1.3 (n = 3)		-
PAS	1.4 ± 0.6 (n = 3)	1.6 ± 0.8 (n = 3)	1.3 ± 0.4 (n = 3)
PAS-R20G	3.6 ± 1.7 (n = 3)	-	-
PAS-N33T	NB (n = 4)	-	-
PAS-E58D	0.6 ± 0.2 (n = 3)	-	-

n is the number of different CM5 chips used to obtain the averaged binding affinities.

Abbreviation: NB, no binding detected for the examined concentrations of analytes.

We also determined the affinity of CNBH/CNBH domain interaction by applying free CNBH domains over the range of concentrations to the immobilized CNBH domains. The SPR response increased with the increase in the free CNBH domain concentration (Fig. 1*D*). Fits of the SPR response indicated that CNBH domains interacted with an averaged affinity of 2.7 ± 1.3 μM. Since the CNBH construct used in our study lacked most of the C-linker responsible for the formation of the “elbow-on-shoulder” interface in the tetrameric C-linker/CNBH domain ring assembly, we expect the affinity of the C-linker/CNBH interdomain interactions in the intact channels to be even higher. Taken together, our results provide the first measurements for the affinities of the PAS/CNBH and CNBH/CNBH domain interactions in the tetrameric PAS/C-linker-CNBH ring assembly in hERG channels.

### F860G/L862G mutations in the intrinsic ligand have no effect on the affinity of the PAS domain interactions with the immobilized CNBH domains

**Figure 2:**
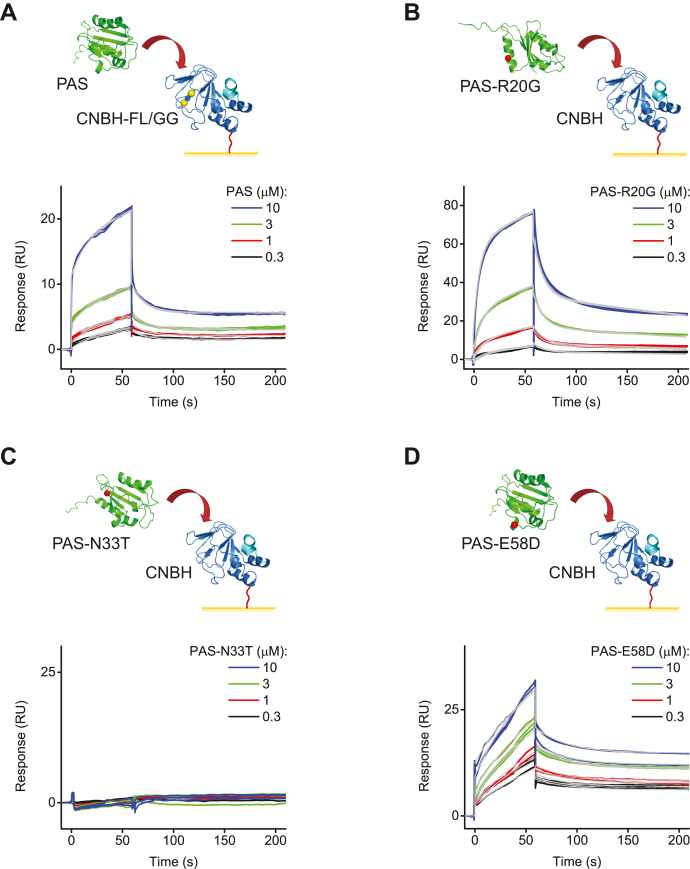
**Effect of mutations in the intrinsic ligand and LQTS-causing mutations on PAS and CNBH domain interactions probed with SPR.***A*, schematic of the hERG PAS domain applied at the indicated concentrations to the hERG CNBH-F860G/L862G mutant domain immobilized on the CM5 sensor chip and representative SPR sensorgrams. Schematic of the mutant hERG PAS domains with R20G (*B*), N33T (*C*), and E58D (*D*) LQTS-causing mutations applied at the indicated concentrations to the hERG CNBH domain immobilized on the CM5 sensor chip and corresponding representative SPR sensorgrams. *Gray lines* represent fits of the data with the two-state reaction binding model using the Biaevaluation software. K_d_ values are 0.6 μM, 2 μM, and 0.2 μM for sensorgrams in (*A*, *B*, and *D*), respectively.

Two recent FRET-based studies suggest that mutations in the intrinsic ligand diminish interactions between PAS and CNBH domains in the full-length channels (37, 38). Especially striking is the effect of the F860G/L862G double mutation in the intrinsic ligand, which abolishes interactions between the PAS and CNBH domains as reflected in the absence of the FRET signal between the N- and C-termini regions tagged with the fluorescence donor and acceptor in the intact hERG channels (37). This is very interesting but also an intriguing result, as in addition to the intrinsic ligand, PAS/CNBH domain interactions also rely on two other interaction networks formed by the PAS-cap/C-linker and diffuse interactions between the PAS and CNBH domain cores. To test if the F860G/L862G mutation is sufficient to abolish interactions between the isolated PAS and CNBH domains, we immobilized CNBH domain with F860G/L862G mutation (F860 and L862 residues are indicated by yellow circles in Fig. 1*B*) and applied free PAS domains over the range of concentrations to the immobilized mutant CNBH-F860G/L862G domains (Fig. 2*A*). The SPR response increased with the increase in the PAS domain concentration. Fits of the SPR response indicated the averaged interaction affinity of 1. 3 ± 0.4 μM for the mutant CNBH and PAS domains. Therefore, our results indicate that at the level of the isolated PAS and CNBH domains, the double mutation in the intrinsic ligand appears to have no effect on the affinity of the interaction between the PAS and CNBH domains.

### Effect of LQTS-causing mutations on the PAS and CNBH domain interactions

Analysis of the interaction interface between the PAS and CNBH domains indicates that the interface harbors several LQTS-causing genetically occurring mutations (14, 24). Some of these mutations, classified as LQT2 class 3 mutations, result in functional channels with altered gating properties (31, 39, 40). Whether these LQTS-causing mutations affect the affinity of the PAS and CNBH domain interactions is not known. Here, we considered R20G, N33T, and E58D class 3 LQTS mutations in the PAS domain, all of which are located at the interface between the PAS and CNBH domains (R20, N33, and E58 residues are indicated by red circles in Fig. 1*B*). To test the effect of the LQTS-causing mutations on the interaction between the PAS and CNBH domains, we immobilized CNBH domains and applied free PAS domains with the LQTS-causing mutations to the immobilized CNBH domains (Fig. 2, *B*–*D*).

It has been shown that R20G and E58D mutations affect hERG channel inactivation, which is thought to be independent of the PAS/CNBH domain interactions, and have no statistically significant effect on the channel voltage dependence and deactivation kinetics (39). Consistent with these functional observations, our SPR-based direct binding results indicate that R20G (Fig. 2*B*) and E58D (Fig. 2*D*) mutations have no statistically significant effect on the binding affinity of the isolated PAS and CNBH domains, as summarized in Table 1 (*p* > 0.3 for the unpaired Student *t* test). Importantly, we found that N33T mutation completely abolished PAS/CNBH domain interactions (Fig. 2*C*). It has been shown that the N33T mutation causes substantial shift in the voltage dependence of hERG channel activation and acceleration of the deactivation kinetics (31, 40). The acceleration of the deactivation kinetics suggests that the N33T mutation weakens PAS and CNBH domain interactions in the full-length hERG channels and supports the absence of interactions between the isolated domains observed with SPR in our study. Therefore, our results for the LQTS mutants show that there is a strong correlation between the effects of the mutations on the binding affinity of the isolated PAS/CNBH domains and the functional effects in the intact channels observed with electrophysiology.

### Oligomeric interactions of PAS domains detected with SPR

**Figure 3:**
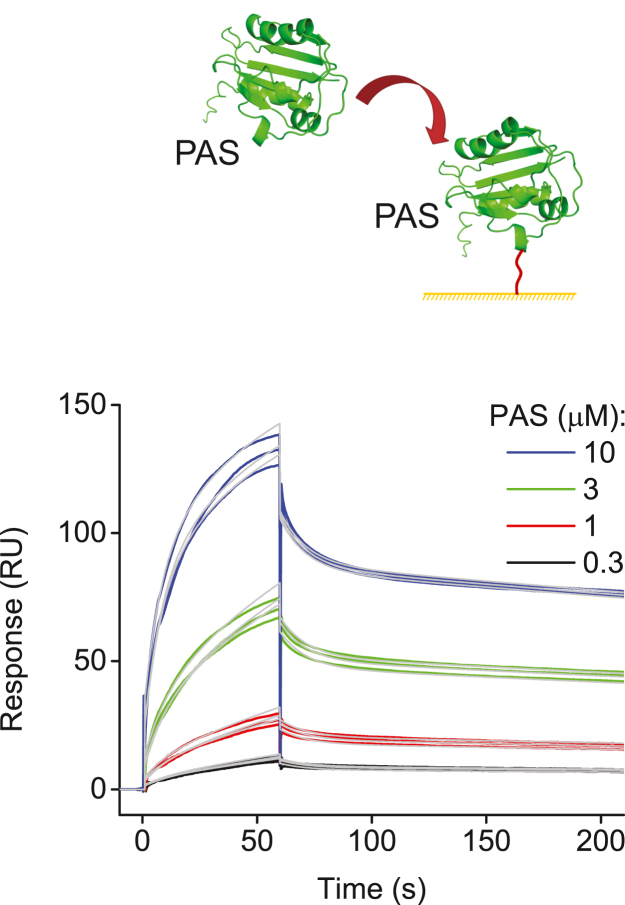
**Oligomerization of the isolated PAS domains detected with SPR.** Schematic of the hERG PAS domain applied at the indicated concentrations to the hERG PAS domain immobilized on the CM5 sensor chip and representative SPR sensorgrams. *Gray lines* represent fits of the data with the two-state reaction binding model using the Biaevaluation software. K_d_ value is 0.4 μM.

PAS domains of hERG channels belong to a large family of proteins and protein modules many of which share low amino acid sequence identity but high structural similarity (41). Eukaryotic PAS domains are known to form homo- and hetero-dimers and promote oligomerization of proteins containing them as modules (41). Therefore, it is not surprising that the chemical cross-linking experiments with glutaraldehyde and analysis of the size-exclusion elusion profile of the hERG PAS domains revealed formation of oligomers (34). To probe the affinity of the PAS domain self-assembly, we immobilized PAS domains on the CM5 sensor chip surface and applied free PAS domains as analytes over the range of concentrations. The SPR response increased with the increase in the PAS domain concentration (Fig. 3). The averaged affinity of PAS/PAS domain interactions was 1.6 ± 0.8 μM, similar to the affinity of PAS/CNBH domain interactions observed with SPR.

Interestingly, application of mEAG PAS domains to the immobilized hERG PAS domains also increased SPR response in a concentration-dependent manner with the affinity of binding of ∼5.6 μM (Fig. S2*A*). Similarly, application of mEAG PAS domains to the immobilized hERG CNBH domains increased SPR response in a concentration-dependent manner with the affinity of binding of ∼1.7 μM (Fig. S2*B*). These results indicate that PAS and CNBH domains of hERG channels can interact not only with PAS domains of hERG channels but also PAS domains from homologous mEAG channels. Although not tested here, this opens a possibility of hERG PAS domain interactions with non-KCNH PAS domains. The promiscuity of these interactions may have far-reaching implications, as discussed in the Discussion section.

### Isolated PAS domains inhibit currents from hERG channels

**Figure 4:**
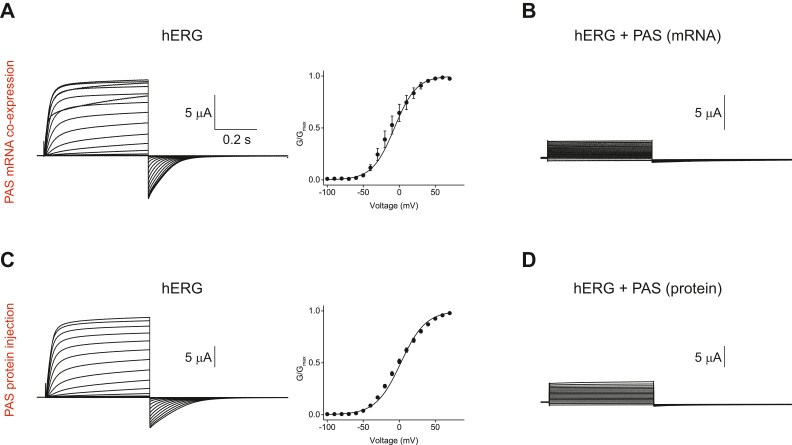
**Isolated PAS domains decrease currents from hERG channels.***A*, representative currents and averaged conductance–voltage relation for hERG channels in the absence of the isolated PAS domains. The *line* represents fit with the Boltzmann equation with the V_1⁄2_ of −6.8 ± 1.3 mV and s of −15.3 ± 0.6 mV. n = 6. *B*, representative currents for hERG channels coexpressed with the isolated PAS domains. The same batch of oocytes was used as in (*A*). Similar results were observed for n = 21 oocytes. *C*, representative currents and averaged conductance–voltage relation for hERG channels in the absence of the isolated PAS domain protein. The *line* represents fit with the Boltzmann equation with the V_1⁄2_ of 2.1 ± 1.2 mV and s of −17.3 ± 0.5 mV. n = 18. *D*, representative currents for hERG channels recorded 3 h after the injection of ∼36 nls of the isolated PAS domain at 40 μM concentration using the Nanoinject II oocyte injector. The same batch of oocytes was used as in (*C*) and different than in (*A*). Similar results were observed for n = 14 oocytes.

The functional effect of the PAS/PAS domain interactions has been explored in several studies before and the results are controversial. Coexpression of hERG channels with the isolated PAS domains in COS cells decreased hERG current density (34). However, other studies reported that coexpression of hERG channels with the isolated PAS domains in *Xenopus laevis* oocytes and HEK cells had no effect on hERG currents (40, 42). Also, a FRET-based study reported that coexpression of CFP-tagged PAS domains with YFP-tagged hERG channels in *Xenopus* oocytes resulted in very weak FRET signal, indicating that the isolated PAS domains do not form strong interactions with full-length hERG channels (43). The coexpression experiments are sensitive to the ratio of the coexpressed proteins. The discrepancy in the findings of the different studies may be due to the differences in the expression levels of the isolated PAS domains relative to the full-length channels. If the isolated PAS proteins are expressed at much lower levels than the full-length hERG channels, the effect of the PAS domains on the currents from hERG channels could be undetectable. Therefore, we repeated the functional experiments by recording currents from oocytes from the same batch divided into two groups. Oocytes in the first group were injected with only mRNA encoding full-length hERG channels at the levels necessary to routinely record medium-size hERG channels. Oocytes in the second group were coinjected with both mRNA encoding the full-length hERG channels (at the same levels as in the first group) and mRNA encoding the isolated hERG PAS domains at 1:250 ratio, which we hoped would ensure overabundance of the isolated PAS domains. While we were able to routinely record medium-size hERG currents in the absence of the isolated PAS domains with the gating properties characteristic of hERG channels for oocytes in the first group (Fig. 4*A*), currents recorded from oocytes in the second group coexpressing hERG and isolated PAS domains had much smaller steady-state activation currents and had no tail currents (Fig. 4*B*). Noteworthy, the steady-state activation currents recorded from oocytes coexpressing full-length hERG channels and isolated PAS domains were substantially larger than the currents detected in oocytes injected with only PAS domain mRNA (Fig. S3).

Our results indicate that coexpression of the isolated PAS domains with full-length hERG channels decreased hERG currents. However, this decrease could be due to the suppression of hERG channel expression by the coinjection of the mRNA encoding the isolated PAS domains by possibly overtaking the protein expression machinery of oocytes, rather than the direct interaction with hERG channels. To test this possibility, we first expressed hERG channels in oocytes and then divided the oocytes into two groups, the control group expressing only hERG channels and the test group where the oocytes expressing hERG channels were injected with ∼36 nls of the purified PAS domain protein at 40 μM concentration. We then compared currents recorded from the two groups of oocytes 3 h after the PAS protein injection. Currents recorded from oocytes in the control group expressing hERG channels in the absence of the isolated PAS protein had typical characteristics of currents recorded from hERG channels (Fig. 4*C*), while currents recorded from hERG channels in the presence of the isolated PAS domain protein were substantially reduced and lacked tail currents (Fig. 4*D*). These results were consistent with the decrease of currents from hERG channels coexpressed with the isolated PAS domains. Taken together, these results suggest that the isolated PAS domains are decreasing currents most likely by interacting with hERG channels.

### Contribution of electrostatic interactions to the PAS/CNBH and PAS/PAS domain binding

**Figure 5:**
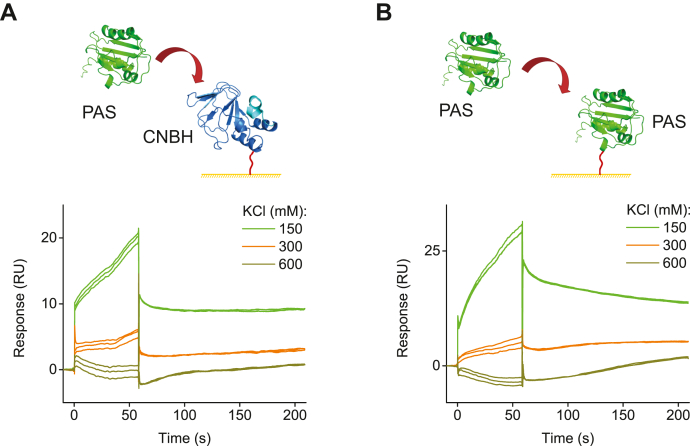
**An electrostatic mechanism contributes to PAS and CNBH domain interactions in hERG channels.** Schematic of the hERG PAS domains applied to the hERG CNBH domains (*A*) and hERG PAS (*B*) domains immobilized on the CM5 sensor chip, and representative SPR sensorgrams. The PAS domains were applied at 3 μM concentration in the buffer containing 1 mM TCEP, 10% Glycerol, 0.05% Tween 20, 30 mM HEPES, pH 7.5, and KCl at the indicated concentrations.

PAS and CNBH domains form an extensive interaction network in hERG channels. Many of the interactions at the interface of the PAS and CNBH domain are electrostatic in mechanism. For instance, it was shown that E56 on the PAS domain forms electrostatic interactions with D803 on the CNBH domain of hERG channels (30). To test the contribution of electrostatic interactions to the affinity of the PAS and CNBH domain binding, we immobilized CNBH domain on the CM5 sensor chip and recorded SPR response for the free PAS domain applied at 3 μM concentration in solutions containing 150, 300, or 600 mM KCl. Increasing ionic strength of the solution gradually decreased the SPR response, with no binding detected in 600 mM KCl (Fig. 5*A*). We also tested the contribution of electrostatic interactions to the PAS domain oligomerization. For these experiments PAS domains were immobilized on CM5 sensor chip and free PAS domains at 3 μM concentration in solutions containing 150, 300, or 600 mM KCl were applied to the immobilized PAS domains. Similar to the effect observed for PAS/CNBH interactions, increasing ionic strength of the solution gradually decreased the SPR response, with no binding detected in 600 mM KCl (Fig. 5*B*). These results indicate that electrostatic interactions play a major role in the PAS/CNBH and PAS/PAS domain binding.

## Discussion

Here, we determined the binding affinities for the isolated hERG PAS and CNBH domain interactions using SPR. We found that the binding affinity for the isolated PAS and immobilized CNBH domains was 1.4 ± 0.6 μM and for the isolated CNBH and immobilized CNBH domains was 2.7 ± 1.3 μM. We found that the R20G and E58D LQTS-causing mutations in the PAS domain had no statistically significant effect on the binding affinity to the immobilized CNBH domains, while N33T LQTS-causing mutation completely abolished the binding between the isolated PAS domains and immobilized CNBH domains. Double mutation F860G/L862G in the intrinsic ligand of the isolated CNBH domain had no statistically significant effect on the binding affinity of the isolated PAS and immobilized CNBH domains. We also found that the isolated PAS domains bound to the immobilized PAS domains with an affinity of 1.6 ± 0.8 μM. Importantly, coexpression of the isolated PAS or injection of the purified PAS domain protein decreased currents recorded from hERG channels.

Our study highlights the suitability of the SPR-based approach for the studies of interdomain interactions in hERG channels. The reproducible concentration-dependent SPR response for the free domains injected over the immobilized domains indicates that the detected SPR signal is specific for the interactions between the free and immobilized domains. The agreement in the observed changes in the binding between the isolated PAS and CNBH domains due to the introduction of the LQTS-causing mutations and increased ionic strength with previous electrophysiology-based findings further strengthens the validity of our approach. For instance, we found that R20G and E58D LQTS-causing mutations did not have a statistically significant effect on the binding of the PAS domains to the immobilized CNBH domains (Fig. 2, *B* and *D*). This is in agreement with the previous report that R20G and E58D mutations do not affect the kinetics of hERG channel deactivation, which is a functional indicator of PAS and CNBH domain interactions in the intact channels (26, 39, 43). We found that N33T LQTS-causing mutation completely abolished interactions between the free PAS domains and immobilized CNBH domains (Fig. 2*C*). This is in agreement with the previous report indicating that N33T mutation accelerates deactivation of hERG currents, suggesting that the mutation decreases interaction between the PAS and CNBH domains in the intact hERG channels. Finally, we also observed that the increase in the ionic strength abolished interactions between the free PAS domains and immobilized CNBH domains (Fig. 5*A*). This finding is consistent with previous reports on the importance of electrostatic interactions for PAS and CNBH domain binding in hERG channels (30).

Overall, our SPR-based results for the interactions of the isolated PAS and CNBH domains are consistent with previous reports; however, there is a notable exception. We found that the double mutation F860G/L862G in the intrinsic ligand has no statistically significant effect on the binding affinity of the free PAS and immobilized CNBH domains (Fig. 2*A*). This is contrary to the findings of two recent studies. One of the studies found that F860G/L862G double mutation in the intrinsic ligand almost completely abolished the FRET signal between the CFP-tagged PAS and Citrine-tagged CNBH domains in the intact hERG channels and, also, drastically accelerated the deactivation kinetics of currents recorded from hERG channels (37). The second study examined the effect of mutations in F860 on the deactivation kinetics and FRET signal between the Cerulean-tagged PAS domain and Venus-tagged CNBH domain in the intact hERG channels (38). Mutations of F860 to hydrophobic residues Val and Ile had no effect on the kinetics of hERG channel deactivation, while mutations to Tyr, Ala, Arg, and Glu substantially accelerated deactivation in hERG channels. In addition, F860R and F860E mutations decreased FRET signal between the donor- and acceptor-tagged PAS and CNBH domains in the intact hERG channels. These two studies suggest that mutations in the intrinsic ligand substantially decrease interactions between the PAS and CNBH domains in intact hERG channels.

How can we reconcile the SPR-based findings with the previous functional and FRET-based reports? For the SPR-based study we employed isolated PAS and CNBH domains, while the electrophysiology and FRET-based studies were performed in the full-length intact hERG channels. The structural alignment of the isolated PAS and CNBH domains shows overall high similarity to the structures of the corresponding domains in the intact hERG channels (14, 26, 35), with RMSD of 1.4 Å for PAS domains and 2.1 Å for CNBH domains (Fig. S1). However, there are also subtle differences in some regions, including the beta strand of the intrinsic ligand that is slightly moved in the isolated CNBH domain structure relative to the structure of this domain in the intact hERG channels (Fig. S1, dashed circle). These subtle differences could contribute to the discrepancy in the results of the SPR-based studies of isolated PAS/CNBH domain interactions and studies based on the examination of PAS/CNBH domain interactions in intact hERG channels. Therefore, it is possible that the intrinsic ligand plays more pivotal role for PAS/CNBH domain interactions in the full-length channels than for the isolated PAS/CNBH domains. While this is a plausible explanation, it is important to mention that the PAS and CNBH domains have an extensive interaction interface in intact hERG channels and the interactions with the intrinsic ligand form only one of the interaction networks (14, 24, 30). The F860G/L862G mutations would still leave intact a network of interactions between the PAS-cap and C-linker and interactions formed by the cores of the PAS and CNBH domains. The CNBH_FL/GG domain used in our SPR-based study lacked most of the C-linker and still showed similar binding to the PAS domain as the wild-type CNBH domain. Therefore, if anything, we would expect stronger binding in intact channels that have additional interaction network between the C-linker and PAS-cap. This argument leads us to propose that F860G/L862G mutation may functionally disengage the PAS and CNBH domains, as reflected in the accelerated deactivation of hERG currents, but does not prevent PAS and CNBH domain binding, as indicated by the SPR-based experiments here. The functional disengagement of the PAS and CNBH domains caused by the F860G/L862G mutation could change the relative position or increase the distance between the acceptor and donor tags, resulting in the decreased FRET signal, which reports on the proximity of the fluorescent tags and not on the direct binding between the tagged PAS and CNBH domains in hERG channels.

We also detected a strong interaction between the isolated hERG PAS domains with SPR (Fig. 3). This interaction was abolished with the increase in the ionic strength (Fig. 5*B*) and was nonspecific for hERG PAS domains, as we also detected binding between the isolated mEAG and hERG PAS domains (Fig. S2*A*). These results indicate that the isolated PAS domain of hERG channels can form homomers and heteromers with PAS domains of other proteins. PAS domains of KCNH channels share structural homology to a vast family of PAS proteins that function as ligand-binding and protein–protein interaction domains and frequently oligomerize (41, 44, 45). The size exclusion profile and chemical cross-linking experiments indicated that the isolated PAS domains of hERG channels also form oligomers (34). Noteworthy, crystal structures of the isolated mEAG and hERG PAS domains indicated a dimeric arrangement of these domains; however, the low conservation in the packing arrangements between the PAS domains of mEAG and hERG channels suggested that the observed assembly is most likely a crystallization artifact (46).

In addition to detecting PAS/PAS domain interactions with SPR, we also report that the isolated PAS domains inhibited currents from hERG channels when coexpressed with full-length hERG channels or injected as purified proteins into oocytes expressing hERG channels (Fig. 4), in agreement with an earlier report that coexpression of the isolated PAS domains and hERG channels in COS cells decreased hERG current density (34). However, these results differ from the results of other studies reporting that the isolated PAS domains do not affect currents from hERG channels when coexpressed with hERG channels in *Xenopus* oocytes and HEK cells (40, 42) and do not interact with the YFP-tagged hERG channels when coexpressed as the isolated CFP-tagged proteins in *Xenopus* oocytes, as reflected in the absence of detectable FRET signal (43). The differences in the results could be due to the differences in the relative expression levels of the isolated domains and the full-length hERG channels. The studies reporting the inhibition of hERG currents by the isolated domains, including our study, most likely have overabundance of the PAS domains, while studies reporting no effect of the isolated domains could have low ratio of the isolated PAS domains relative to the full-length channels. Taken together, our SPR-based and electrophysiology results suggest that the isolated PAS domains interact with each other and inhibit currents from hERG channels.

**Figure 6:**
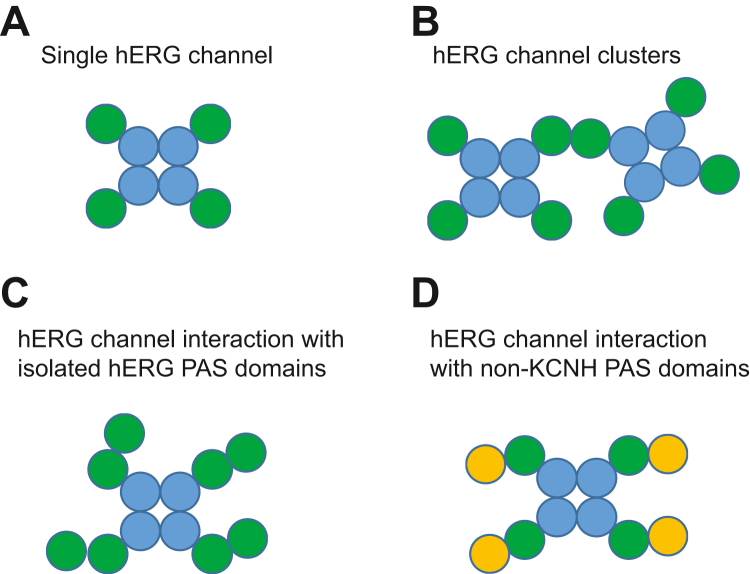
**Potential mechanisms of PAS/PAS domain interactions.***A*, schematic of a single hERG channel. PAS domains are shown in *green circles* and CNBH domains are shown in *blue circles*. Transmembrane domains are omitted for clarity. *B*, schematic of two hERG channels linked *via* their PAS domains in a potential hERG channel “cluster”. *C*, schematic of the potential interactions between the isolated PAS domains and the PAS domains in the full-length hERG channels. *D*, schematic of the potential interactions between the PAS domains in the full-length hERG channels and other non-KCNH PAS domains shown in *yellow circles*.

The implications of the PAS/PAS domain interactions for the physiological functions of hERG channels could be far reaching. Due to the location of the PAS domains in the structure of intact hERG channels at the periphery of the intracellular tetrameric ring assembly (Fig. 1*B*) (14), the PAS/PAS domain interactions will not be occurring within a single hERG channel (Fig. 6*A*). However, there are several physiological and pathological situations when the PAS/PAS interactions could become important. It has been well documented that hERG channels are overexpressed in cancer (47, 48). The increased surface density of hERG channels in cancer could place multiple hERG channels close to each other, leading to formation of hERG channel clusters linked by interactions between PAS domains from different adjacent channels (Fig. 6*B*). It has been shown that some LQTS mutations introduce premature stop codon in the N-terminal region of hERG channels (49, 50, 51, 52). Although it is expected that most of the mRNA containing the premature stop codon will be eliminated *via* the nonsense-mediated mRNA decay mechanism (53), it is possible that some of the isolated PAS domains and PAS domain containing regions will still be expressed. In heterozygous individuals, these isolated domains could interact with full-length hERG channels causing hERG current inhibition (Fig. 6*C*). Finally, our results suggest that the PAS/PAS interactions are not specific. Therefore, PAS domains of hERG channels could interact with PAS domains of other proteins (Fig. 6*D*), forming heteromers as observed for many structurally similar PAS proteins (36). Future studies will reveal if and how the proposed PAS/PAS interaction scenarios contribute to the hERG-channel-mediated cell signaling.

## Experimental procedures

### Protein expression and purification

DNA encoding wild-type and mutant PAS (residues 2–134) and CNBH (residues 734–867) domains of hERG channels (GI # Q12809) and PAS domains (residues 7–136) of mEAG channels (GI # Q60603) was synthesized by BioBasic and subcloned into pETM11 bacterial expression vector containing an N-terminal 6-His affinity tag followed by a tobacco etch virus (TEV) protease cleavage site. The DNA sequences were verified by sequencing (Genewiz). The PAS and CNBH domains were expressed in BL21 (DE3) *Escherichia coli* cells as previously described (54, 55). The cells were grown at 37 °C to an optical density at 600 nm of 0.6 to 0.8, induced with IPTG at 18 °C overnight and harvested by centrifugation. The cells were resuspended in 150 mM KCl, 1 mM TCEP, 1 mM ABSF, 2.5 mg/ml DNaseI, and 30 mM HEPES, pH 7.5. Cells were lysed with an Emulsiflex-C5 (Avestin). Insoluble protein was separated by centrifugation in 45 Ti rotor at 30,000 rpm for 1 h at 4 °C. The PAS and CNBH domains were purified by Ni^2+^ affinity chromatography using HisTrap HP column (GE Healthcare) and eluted on a linear gradient to 500 mM imidazole. The 6-His tag was cleaved with TEV protease. The protein was further purified on a Superdex 200 Increase 10/300 column (GE Healthcare) equilibrated with 150 mM KCl, 1 mM TCEP, 10% glycerol, and 30 mM HEPES, pH 7.5. The protein concentration was determined with Bradford Protein Assay Kit (Pierce).

The purified protein was stored at −80 °C in aliquots and thawed immediately before the experiments. The molecular weight of the PAS and CNBH domains used in the study was verified on Coomassie-Blue-stained gels and with mass spectrometry at Proteomics and Metabolomics Core Facility at Georgetown University Medical Center.

### Surface plasmon resonance measurements

All SPR binding experiments were performed on a CM5 chip (GE healthcare) at 25 °C using a Biacore T200 Instrument (GE Healthcare). The purified PAS and CNBH domains were immobilized on the CM5 chip (GE Healthcare) using a standard amine coupling chemistry in the presence of 10 mM sodium acetate buffer at pH 5.5 as the immobilization buffer (buffer used to directly dissolve ligands). HBS-P buffer (150 mM NaCl, 10 mM HEPES, 0.05% (v/v) surfactant P20, pH 7.4) was used as the immobilization running buffer (buffer that runs in the background during immobilization). Proteins were then injected at 200 nM concentrations and immobilized at ∼2000 to 3000 RU (1 RU = 1 pg of protein per mm^2^). For all experiments, a reference flow cell (FC) was activated and deactivated, using the same standard amine coupling chemistry, as in active FC with immobilized proteins, but without protein. This reference FC was used as a reference surface to account for a potential nonspecific binding to the chip surface. The SPR signals corresponding to the reference FC were subtracted from the ones corresponding to the surfaces with immobilized proteins (active FCs). In addition, the binding corresponding to blank injections (buffer only) was subtracted from the reference subtracted SPR data.

A running buffer containing 150 mM KCl, 1 mM TCEP, 10% Glycerol, 0.05% Tween 20, 30 mM Hepes, pH 7.5 was used in all SPR direct binding experiments between analytes (free PAS or CNBH domains) in solution and immobilized proteins, unless specified. Analytes were injected over the range of concentrations over the chip surface in triplicates for 60 s at a flow rate of 50 μl/min (association phase), followed by buffer only injections for 150 s (dissociation phase). Injection of Glycine (pH 2.0) for 15 s was used to regenerate the chip surface between the analyte injections. To determine the binding affinity (K_d_), the SPR sensorgrams were first fitted with a simple 1:1 binding model using Biaevaluation software version 1.0. However, the 1:1 model failed to predict the data (Fig. S4*A*), indicating that the binding mechanism is more complex. We then followed the procedures outlined in the previous publication (36) to search for a possible underlying biphasic interaction mechanism, which predicted a biphasic two-step conformational change interaction mechanism. Based on this manual fitting prediction for some representative SPR profiles, we used two-state reaction model available in the Biaevaluation software version 1.0 to fit the sensorgrams. The two-state reaction model adequately predicted the SPR sensorgrams (Fig. S4*B*).

The nonspecific injection signal at the beginning of the association and dissociation phases was excluded from data fitting and from the figures. Each of the SPR-based experiments was repeated at least three times on three different CM5 chips. The error bars on the figures and Table 1 correspond to the S.E.

### Electrophysiology

The cDNA encoding hERG1-S631A in pGH19 vector was kindly provided by G. Robertson (University of Wisconsin-Madison, Madison, WI). For the isolated PAS domain expression, cDNA of the isolated PAS domain was subcloned into pGH19 vector. The mRNA was transcribed using the T7 mMessage mMachine kit (Thermo Fisher Scientific). Defolliculated *Xenopus laevis* oocytes were purchased from Ecocyte Bioscience and injected with the mRNA using a Nanoinject II oocyte injector (Drummond). Each batch of oocytes was harvested from one frog. The experiments were repeated in oocytes harvested from three different frogs.

For current recordings, oocytes were placed into a RC-3Z chamber (Warner Instruments). The currents were recorded using Two-Electrode Voltage Clamp (TEVC) technique with OC-725C amplifier (Warner Instruments) and pClamp11 software (Molecular Devices). The signals were digitized using Digidata 1550 (Molecular Devices). Microelectrodes were pulled from borosilicate glass and had resistances of 0.7 to 1.5 M when filled with 3 M KCl. The recording (bath) solution contained 96 mM NaCl, 4 mM KCl, 0.1 mM CaCl2, 1.8 mM MgCl2, and 5 mM HEPES, pH 7.5. The hERG currents were elicited by applying a series of 0.1-s voltage pulses ranging from −100 to +70 mV in 10 mV increments from a holding potential of −80 mV, followed by a 0.15-s voltage pulse to −100 mV. The currents were not leak-subtracted.

To analyze voltage dependence of the tail currents, peak tail-current amplitudes were normalized to the largest peak tail-current amplitude (Gmax). These normalized data were then plotted against the test voltage and were fitted with a Boltzmann equation,

$$G/G_{max}=\frac{1}{1+e^{\left(\frac{V-V_{1/2}}{s}\right)}}$$

where *V* represents the test voltage (mV), *V*_1⁄2_ is the midpoint activation voltage (mV), and *s* is the slope of the relation (mV).

## Data availability

All data are contained with the article or available on request by contacting the corresponding author: tib5@georgetown.edu.

## Supporting information

This article contains supporting information.

## Conflict of interest

The authors declare that they have no conflicts of interest with the contents of this article.
