# Supplementary material for: Investigation of PAS and CNBH domain interactions in hERG channels and effects of long-QT syndrome-causing mutations with surface plasmon resonance
Source: J Biol Chem. 2021 Nov 19;298(1):101433. doi: 10.1016/j.jbc.2021.101433 (PMC8693265; doi:10.1016/j.jbc.2021.101433)
Supplement: Supplemental Figure S1-S4 [file mmc1.pdf]

Supporting Information

Investigation of PAS and CNBH domain interactions in hERG channels and effects of long-QT syndrome-causing mutations with surface plasmon resonance

Stephanie M. Soohoo[1#], Purushottam B. Tiwari[2#], Yuichiro J. Suzuki[1], Tinatin I. Brelidze[1*]

[1]Department of Pharmacology and Physiology, Georgetown University Medical Center, Washington, DC, 20057, USA
[2]Department of Oncology, Georgetown University Medical Center, Washington, DC, 20057, USA

Running title: Investigating hERG PAS and CNBH interactions with SPR

[#] These authors contributed equally

* To whom correspondence should be addressed: Tinatin I. Brelidze, Department of Pharmacology and Physiology, Georgetown University Medical Center, Washington, DC, 20057, USA; tib5@georgetown.edu; Tel: (202)-687-6178

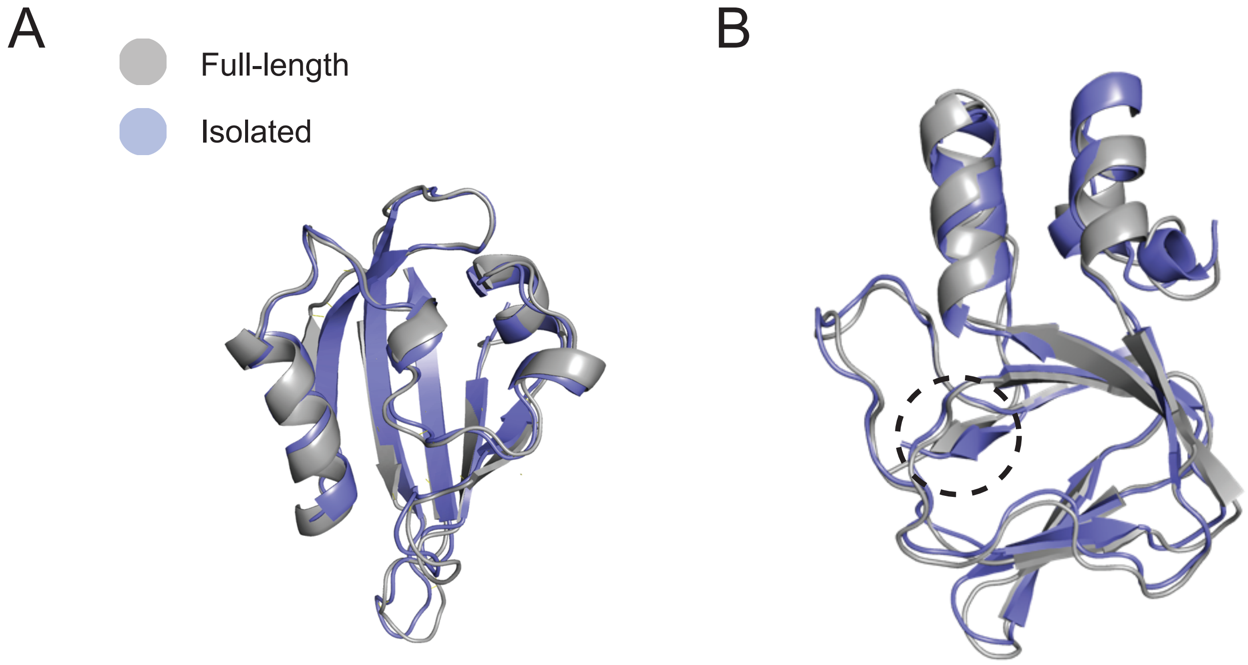

Figure S1. Structures of the isolated PAS and CNBH domains are similar to the PAS and CNBH domain structures from the full-length hERG channels. A, Structural alignment of the isolated PAS domains (purple, PDB ID 1BYW) and PAS domains from the full-length hERG channel structure (grey, 5VA2). RMSD of 1.4 Å. B, Structural alignment of the isolated CNBH domains (purple, PDB ID 6SYG) and CNBH domains from the full-length hERG channel structure (grey, 5VA2). RMSD of 2.1 Å. The intrinsic ligand is circled by a dashed line.

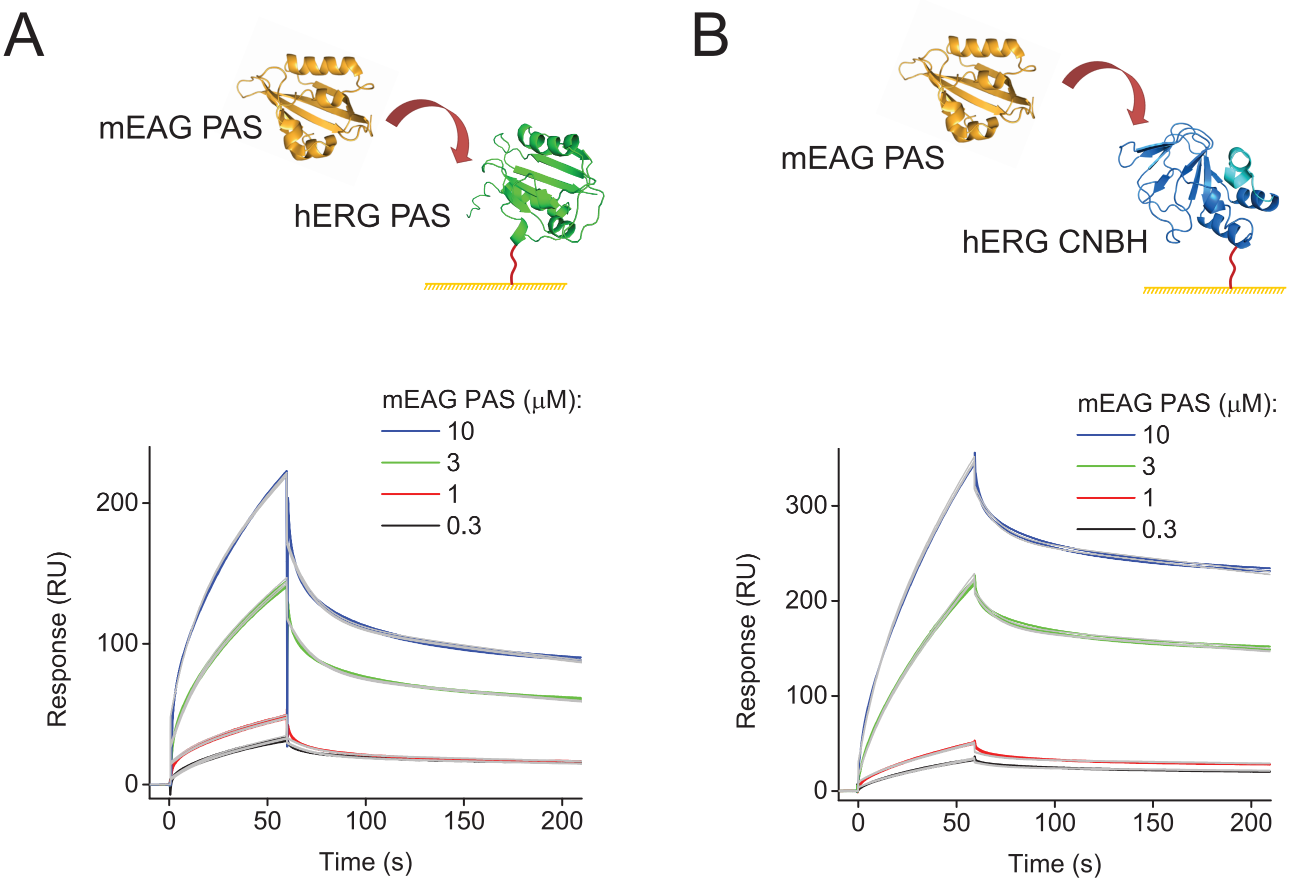

Figure S2. mEAG PAS domains interact with hERG PAS and CNBH domains. Schematic of the mEAG PAS domains applied at the indicated concentrations to the hERG PAS (A) and hERG CNBH (B) domains immobilized on the CM5 sensor chip and representative SPR sensorgrams. Grey lines represent fits of the data with the two state reaction binding model using the Biaevaluation software. K_d values are 5.6 μM and 1.7 μM for sensorgrams in (A) and (B), respectively.

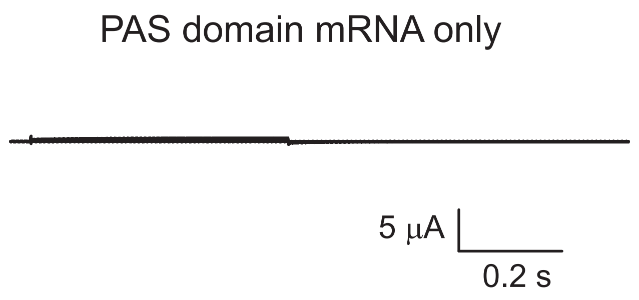

Figure S3. Expression of the isolated PAS domain in oocytes does not generate noticeable currents. Representative currents recorded from oocytes expressing only the isolated PAS domains. The same batch of oocytes was used as for experiments in Figs. 4A and 4B, and the same amount of the PAS domain mRNA was injected as for Fig. 4B. Similar results were observed for n = 7 oocytes.

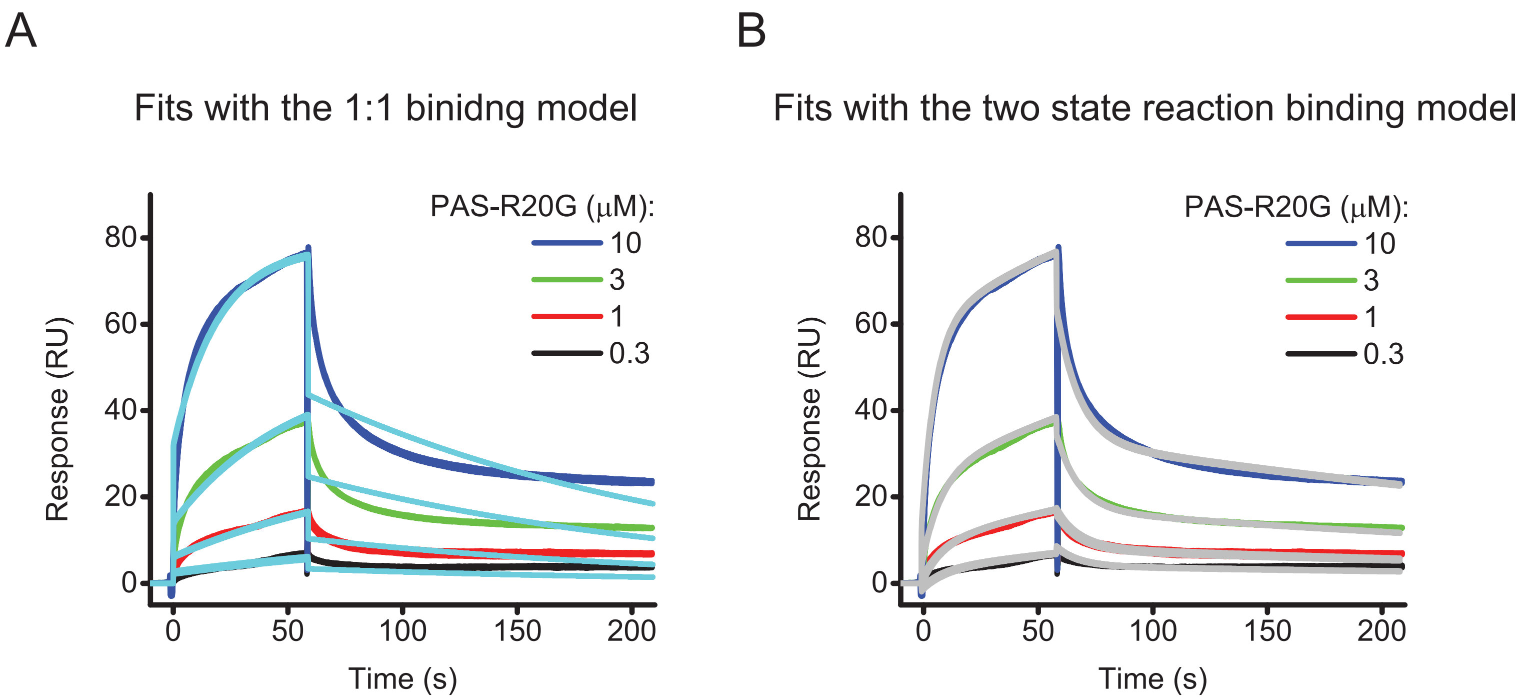

Figure S4. Comparison of fits with 1:1 and two-state binding models. SPR sensorgrams for the R20G mutant PAS domains applied to the immobilized CNBH domains at the indicated concentrations fitted with the 1:1 model (A, cyan lines) and two state reaction binding model (B, grey lines). The SPR sensorgrams are the same as shown in the main Fig. 2B. Biaevaluation software version 1.0 was used for fitting.
